# Diagnosis and treatment of iron deficiency anemia during pregnancy and the postpartum period: Iron deficiency anemia working group consensus report

**DOI:** 10.4274/tjod.01700

**Published:** 2015-09-15

**Authors:** Olus Api, Christian Breyman, Mustafa Çetiner, Cansun Demir, Tevfik Ecder

**Affiliations:** 1 Yeditepe University Hospital, Clinic of Gynecology and Obstetrics, İstanbul, Turkey; 2 Zurich University Hospital, Feto Maternal Hematology Unit, Zurich, Switzerland; 3 Koç University Faculty of Medicine American Hospital, Department of Hematology, İstanbul, Turkey; 4 Çukurova University Faculty of Medicine, Department of Gynecology and Obstetrics, Adana, Turkey; 5 İstanbul Bilim University Faculty of Medicine, Department of Internal Medicine, Division of Nephrology, İstanbul, Turkey

**Keywords:** Iron deficiency anemia, Pregnancy, postpartum period, intravenous iron therapy

## Abstract

According to the World Health Organization (WHO), anemia is the most common disease, affecting >1.5 billion people worldwide. Furthermore, iron deficiency anemia (IDA) accounts for 50% of cases of anemia. IDA is common during pregnancy and the postpartum period, and can lead to serious maternal and fetal complications. The aim of this report was to present the experiences of a multidisciplinary expert group, and to establish reference guidelines for the optimal diagnosis and treatment of IDA during pregnancy and the postpartum period. Studies and guidelines on the diagnosis and treatment of IDA published in Turkish and international journals were reviewed. Conclusive recommendations were made by an expert panel aiming for a scientific consensus. Measurement of serum ferritin has the highest sensitivity and specificity for diagnosis of IDA unless there is a concurrent inflammatory condition. The lower threshold value for hemoglobin (Hb) in pregnant women is <11 g/dL during the 1^st^ and 3^rd^ trimesters, and <10.5 g/dL during the 2^nd^ trimester. In postpartum period a Hb concentration <10 g/dL indicates clinically significant anemia. Oral iron therapy is given as the first-line treatment for IDA. Although current data are limited, intravenous (IV) iron therapy is an alternative therapeutic option in patients who do not respond to oral iron therapy, have adverse reactions, do not comply with oral iron treatment, have a very low Hb concentration, and require rapid iron repletion. IV iron preparations can be safely used for the treatment of IDA during pregnancy and the postpartum period, and are more beneficial than oral iron preparations in specific indications.

## INTRODUCTION

According to the World Health Organization (WHO), anemia affects approximately 1.5 billion people worldwide. The prevalence is very high in Africa, Asia, India, Latin America, Eastern Europe, and China; however, it is also high in developed countries^(1,2)^.

Anemia has the highest prevalence in 3 groups: children aged <5 years (47%), pregnant women (42%), and women of reproductive age (30%). Iron deficiency is seen in 50% of cases and is the most common cause of anemia^(1,2)^. No national epidemiologic study on the prevalence of anemia has been conducted in Turkey but some regional studies have been performed^(3,4,5,6,7,8,9,10)^. The WHO placed Turkey on the worldwide anemia map by extrapolating these data. Accordingly, Turkey is in the intermediate group, with a prevalence of anemia between 20% and 39.9% among women of reproductive age, and is in the severe group for pregnant women, with a prevalence of 40%^(1,2)^.

Attention has focused on the alternative use of intravenous (IV) iron preparations because oral iron therapy has some disadvantages^(11,12,13)^. Many studies on IV iron sucrose and ferric carboxymaltose have been conducted. Data on other IV iron preparations such as iron gluconate, iron sorbitol, iron polymaltose, iron isomaltoside, and low molecular weight iron dextran are very limited, and there are increasing safety concerns about high molecular weight iron dextran, which is no longer available in Europe due to a high rate of serious adverse events.

Recent guidelines published in England, Switzerland, Germany, and Asia-Pacific have included IV iron therapy, and the Network for Advancement of Transfusion Alternatives (NATA) also published reports on the indications for IV iron therapy^(14,15,16,17,18)^.


Recommendations• Large-scale, multicenter studies should be performed to accurately collect population-based data on iron deficiency anemia in Turkey.


## MATERIALS AND METHODS

The members of the working group reviewed the studies and guidelines on the diagnosis and treatment of iron deficiency anemia published in Turkish and international journals. About 200 literature articles were screened. Search terms were iron deficiency anemia, pregnancy, postpartum period, and parenteral iron therapy. Sources about iron deficiency prophylaxis and oral iron therapy were excluded because the focus was iron deficiency anemia and parenteral iron therapy. The included sources were listed in references part. With the data from the reviewed literature and the working group’s own experiences, conclusive recommendations were made as a scientific consensus.

What follows is a review of the diagnosis of iron deficiency anemia and intravenous iron therapy; prophylaxis and oral iron therapy will not be discussed.

## IRON DEFICIENCY ANEMIA DURING PREGNANCY

### Diagnosis

The major signs and symptoms of iron deficiency anemia can be summarized as fatigue, low physical and mental capacity, headache, vertigo, leg cramps, pagophagia, cold intolerance, koilonychias, mucosal paleness, and angular stomatitis. Iron deficiency anemia during pregnancy poses a number of maternal and fetal problems, including premature birth, intrauterine developmental retardation, placental problems, a decrease in newborn iron storage, the risk of a decrease in maternal blood reserves during birth, and the need for transfusion in cases of heavy blood loss, cardiac stress, symptoms of anemia, prolonged hospital stay, decreased maternal breast milk production, and maternal depletion of iron stores during and after the postpartum period. As such, diagnosis and effective treatment of iron deficiency anemia are of critical importance^(19,20,21,22)^.

The most important factor in the diagnosis of iron deficiency anemia is laboratory testing. The classic laboratory findings of iron deficiency anemia include a decrease in the hemoglobin (Hb) level, serum iron concentration, serum transferrin saturation, and serum ferritin level, and an increase in total iron-binding capacity. In fact, it is adequate to study the complete blood count and serum ferritin for diagnosis. A serum ferritin concentration <30 μg/L together with an Hb concentration <11 g/dL during the 1^st^ trimester, <10.5 g/dL during the 2^nd^ trimester, and <11 g/dL during the 3^rd^ trimester are diagnostic for anemia during pregnancy. Measurement of the serum ferritin concentration is the most accurate test in patients without underlying inflammation, and a serum ferritin level below the threshold value alone is adequate for diagnosis in the absence of other tests; however, physicians should be aware that serum ferritin is also an acute phase reactant and may be normal, even elevated, under inflammatory conditions despite the presence of anemia, and in such cases confirmation of the diagnosis may require additional tests.

It is recommended to measure serum ferritin at least once early in pregnancy. If ferritin and hemoglobin indicate iron deficiency anemia, anemia treatment should be initiated (note that intravenous iron is not warranted for the use in first trimester); if ferritin and hemoglobin levels are normal, prophylactic oral iron therapy should be commenced. It is not necessary to measure serum ferritin again later in pregnancy unless the symptoms of anemia occur. On the other hand, Hb should be measured in each trimester because the probability of an increase in the need for iron and development of iron deficiency is always possible, even if the baseline value is normal. Moreover, the Hb concentration during delivery is important because a low maternal Hb can result in fetal problems, including mortality^(1,17,23,24,25,26,27)^.

**Figure 1:**
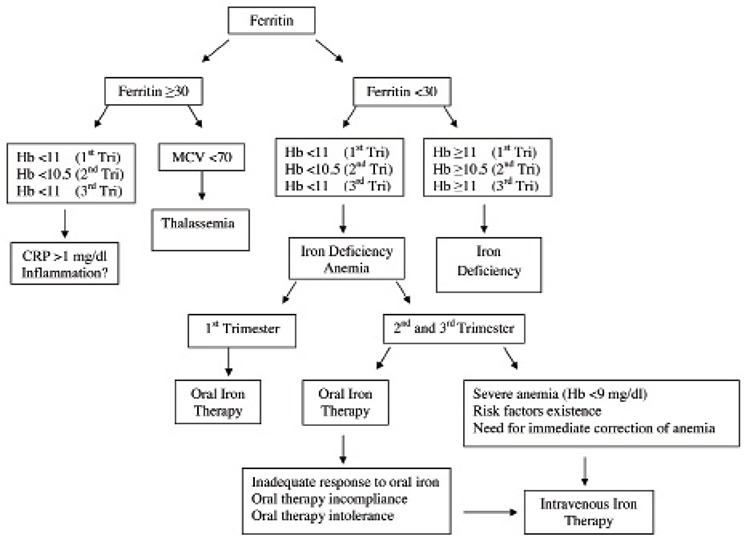
Algorithm for the diagnosis and treatment of iron deficiency anemia during pregnancy

If serum ferritin is low (<30 μg/L), but the Hb is normal (≥11 g/dL during the 1^st^ trimester, ≥10.5 g/dL during the 2^nd^ trimester, and ≥11 g/dL during the 3rd trimester) the diagnosis is iron deficiency; however, if serum ferritin is low (<30 μg/L) and Hb is also low (<11 g/dL during the 1^st^ trimester, <10.5 g/dL during the 2^nd^ trimester, and <11 g/dL during the 3^rd^ trimester), the diagnosis is iron deficiency anemia. When Hb is low (<11 g/dL during the 1^st^ trimester, <10.5 g/dL during the 2^nd^ trimester, and <11 g/dL during the 3^rd^ trimester), but serum ferritin is normal (≥30 μg/L) additional tests, such as transferrin saturation, serum iron, total iron-binding capacity, and C-reactive protein (CRP), are needed for diagnosis. When serum ferritin is normal (≥30 μg/L), but mean corpuscular volume (MCV) is low (<70 fL) in the absence of inflammation, the diagnosis might be thalassemia and further investigation is required (Figure 1).


Recommendations• The serum ferritin level, which is the most sensitive test at baseline, should be measured together with the Hb level to diagnose iron deficiency. A serum ferritin level <30 μg/L during pregnancy should prompt treatment. Monitoring in further periods should be based on the Hb concentration, which should be measured in each trimester.


## TREATMENT

As the need for iron increases during pregnancy, prophylactic oral iron therapy is given to all pregnant women with normal laboratory values; however, the primary cause of morbidity is iron deficiency anemia. Data suggesting that anemia causes cardiovascular diseases in further stages of life are increasing. Oral iron preparations can be used throughout pregnancy, whereas IV iron therapy is recommended during the 2^nd^ and 3^rd^ trimesters. There are some instances for which switching to an IV iron preparation is advantageous (Table 1). Actually, IV iron therapy should be the first option in the presence of severe anemia and risk factors, and in emergency situations, because IV therapy is more effective and rapid than oral therapy for resolving anemia^(1,17,26,28,29,30)^.

Threshold values for Hb that indicate oral or IV iron therapy during pregnancy according to countries and guidelines:


PregnancyOral ironIV ironSwitzerland9< Hb ≤10.5 g/dLHb ≤9 g/dLGermany9< Hb ≤11.5 g/dLHb ≤9 g/dLAsia-Paci c10< Hb ≤10.5 g/dLHb ≤10 g/dLNATAHb ≤11 g/dL (2^nd^ Trimester)Hb ≤11 g/dL (3^rd^ Trimester)


Hb threshold values can be higher in the presence of additional risk factors, such as coagulation disorders and placenta previa. The reason that Hb threshold levels are higher in Asia-Pacific countries is that there the goal of reducing the need for blood transfusion is achieved via IV iron therapy because blood transfusion cannot be performed frequently^(14,15,16,17,18)^.


Recommendations• IV iron therapy should be considered from the 2^nd^ trimester onwards in pregnant women with iron deficiency anemia that cannot tolerate or do not respond to oral iron therapy.• With severe anemia (Hb ≤9 g/dL), the presence of risk factors (such as coagulation disorders, placenta previa) and conditions that require prompt resolution of anemia (paleness, tachycardia, tachypnea, syncope, heart failure, respiratory failure, angina pectoris, and signs of cerebral hypoxia) are other potential indications for IV iron therapy.• The IV iron therapy dose should be individual patient based and bringing the Hb level up to at least 11 g/dL should be the target of the therapy.• Switching from oral to IV iron therapy or starting IV therapy initially is contingent upon risk-benefit assessment; however, such assessment should be performed on an individual patient basis and requirements should be evaluated carefully.



PregnancyOral ironIV ironTurkey9<Hb≤11 g/dL (1^st^ and 3^rd^ trimester) - 9<Hb≤10.5 g/dL (2^nd^ trimester)Hb≤9 g/dL


## IRON DEFICIENCY ANEMIA DURING THE POSTPARTUM PERIOD

**Figure 2:**
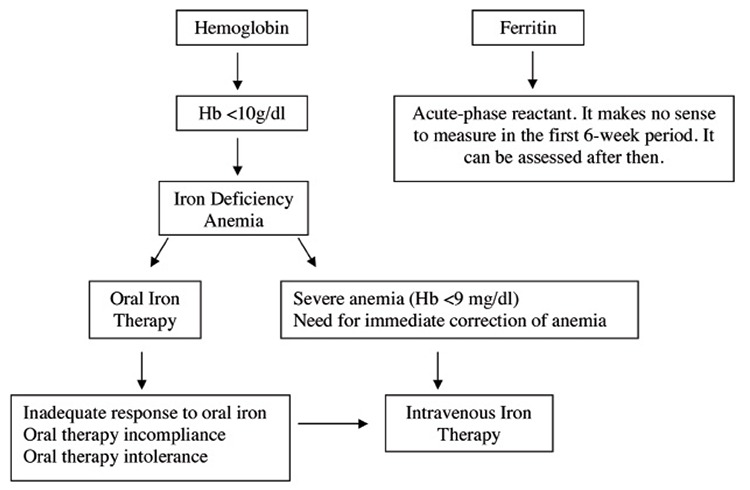
Algorithm for the diagnosis and treatment of iron deficiency anemia during the postpartum period

Postpartum anemia occurs primarily due to inadequate iron intake before and during pregnancy, and blood loss during delivery. In other words, the combination of iron deficiency anemia and hemorrhagic anemia leads to postpartum anemia. Postpartum anemia has been associated with depression, stress, anxiety, cognitive impairment, decreased mother-infant attachment, and infant developmental retardation. The Hb concentration must be checked in patients with excessive blood loss during delivery and/or in those with puerperal symptoms of anemia. A postpartum Hb concentration ≤10 g/dL indicates clinically significant anemia. Moderate-to-severe anemia is considered when Hb is between 9-10 g/dL and an Hb concentration ≤9 g/dL is considered severe anemia^(17,18,27,31,32,33,34)^. It is not meaningful to measure the serum level of ferritin, an acute phase reactant, because it can be normal or elevated during the first 6 weeks post delivery (Figure 2).


Recommendations• The Hb concentration must be checked within 24-48 h after delivery in cases of blood loss >500 mL during the postpartum period, untreated anemia during the antenatal period, or symptoms of anemia during the postnatal period.


## TREATMENT

Treatment is based on the severity of anemia and health status of the puerperal. As a rule, oral iron therapy is recommended in cases of mild anemia; however, it can be switched to IV iron therapy in patients with moderate and severe anemia or in those who cannot tolerate oral iron therapy. After achieving the target values, maintenance therapy can be administered using oral iron preparations. Many studies have reported that IV iron administration is more advantageous than oral administration^(12,13,17,35,36,37)^. Heavy blood loss can occur in women who begin delivery while anemic, and blood transfusion becomes necessary when the Hb concentration drops to 7 g/dL. One study reported that 18% of women who were hospitalized for anemia and postpartum bleeding received transfusion. Another advantage of IV iron therapy is that it reduces the need for blood transfusion. Blood transfusion is in essence a type of transplantation and is associated with serious safety risks, high costs, and availability issues. Transfusion-induced sensitization is another risk for the future. Additionally, IV iron therapy is associated with a shorter duration of hospitalization^(38,39,40,41,42)^.

Various Hb threshold values that indicate oral iron and IV iron therapy during the postpartum period:


PostpartumOral ironIV ironSwitzerland10< Hb ≤11.5 g/dLHb ≤10 g/dLGermany8< Hb≤ 10 g/dLHb ≤8 g/dLAsia-Pacific10< Hb≤ 10.5 g/dLHb ≤10 g/dLNATA-Hb ≤10 g/dL


In Germany, the Hb threshold is <8 g/dL, which is below the limit of severe anemia. This value is extremely low and is probably related to economic reimbursement issues^(14,15,16,17,18)^.

Heavy menstrual bleeding is another condition that can require blood transfusion. Although a precise definition of this clinical condition is lacking, according to the National Institute for Health and Care Excellence (NICE) guidelines, it corresponds to a volume of bleeding that causes health problems and social discomfort, roughly 80 mL/per period. Here too, an indication for IV iron therapy can be mentioned because such patients face the risk of transfusion and this process is likely to progress to hysterectomy^(43,44,45)^.


Recommendations• In patients with an Hb level ≤10 g/dL who are hemodynamically stable and asymptomatic or mildly symptomatic, oral iron 100-200 mg/day should continue for up to 3 months, and complete blood count and serum ferritin values should be checked at the end of treatment.• IV iron therapy should be considered in patients who cannot tolerate or do not respond to oral iron therapy, and have significant symptoms or moderate-severe/severe anemia.• The physician should start IV iron therapy whenever the Hb value is less than or equal to 9 because this is considered as severe anemia.• The therapeutic iron dose should be determined on an individual patient basis and should achieve an Hb level ≥11 g/dL.



PostpartumOral ironIV ironTurkey9< Hb ≤11 g/dLHb ≤9 g/dL


## PARENTERAL IRON THERAPY OPTIONS

Worldwide, iron deficiency anemia is most commonly treated with oral iron preparations; however, oral preparations are associated with problems such as intolerability, poor patient compliance, insufficient response to treatment, and prolonged treatment. Therefore physicians are increasingly interested in parenteral iron therapy options.

## INTRAMUSCULAR IRON THERAPY

Some preparations such as iron sorbitex, high-molecular-weight iron dextran, and low-molecular-weight iron dextran can be given intramuscularly (IM). However, IM injections have some significant drawbacks, therefore IM is generally not recommended. Intramuscular injection is painful, has a risk of permanent skin staining, and is associated with the development of sterile abscesses and gluteal sarcomas. Also, intramuscular absorption of iron is slow, the use of iron given via this route is variable, and IM injections are not possible for patients who have reduced muscle mass. IM administration of iron is not safer or less toxic than the IV route, so the most appropriate parenteral route is IV^(46,47,48,49,50)^.

## INTRAVENOUS IRON THERAPY

IV iron therapy is not associated with oral and IM iron therapy problems and is a more cost-effective option. IV preparations are more expensive than oral preparations, but the total cost of oral treatment becomes equivalent to IV treatment because repeated administrations are needed in oral treatment. Additionally, the patient has the initiative in oral administration and that makes it difficult to monitor how many doses the patient has actually received; if the patient is not receiving the tablets, the physician prescribes more and more oral preparations to treat the patient so the cost increases and becomes even more than IV treatment. The risk of premature birth, the most serious risk associated with anemia during pregnancy, increases in patients with anemic who are not treated. The cost of care for a premature baby far exceeds that of IV iron treatment, which is why interest in IV iron therapy is gradually increasing^(50,51,52,53)^.

## HIGH-MOLECULAR-WEIGHT IRON DEXTRAN

Safety concerns that physicians have regarding parenteral iron preparations are related to serious adverse events (anaphylaxis, shock, and death) reported in association with high-molecular-weight iron dextran, which has been removed from the market in Europe and replaced by newer preparations that have more favorable safety profiles^(54,55,56,57,58,59)^.

## LOW-MOLECULAR-WEIGHT IRON DEXTRAN

There are a limited number of studies on the use of low-molecular weight iron dextran during pregnancy and the postpartum period. No serious adverse events have been associated with its use, but mild adverse events have been observed in 5% of cases. It is contraindicated during the 1^st^ trimester, and the Food and Drug Administration (FDA) pregnancy category is C for the 2^n^d and 3^rd^ trimesters. Data on its use during the neonatal period is lacking and its effect on lactation is unknown. It can be administered via IV injection or infusion. A test dose is required before each IV administration^(18,60,61)^.

## FERROUS GLUCONATE

Ferrous gluconate is not available in Turkey. There few studies on its use during pregnancy and there are data concerning its use during the neonatal period. It is contraindicated during the 1^st^ trimester, and its pregnancy category is B for the 2^nd^ and 3^rd^ trimesters. No serious adverse events have been associated with its use. It can only be administered via the IV route. The maximum single dose is 125 mg and a test dose is not required. Its molecular stability is low. Free iron release can occur and may cause liver necrosis^(62,63)^.

## IRON POLYMALTOSE

There are a limited number of studies on the use of iron polymaltose during pregnancy and the neonatal period, but no serious adverse events have been reported. Its administration is limited to the IM route^(64,65,66,67)^.

## IRON SORBITOL

Iron sorbitol is not available in Turkey. There are few studies on its use during pregnancy and no data exists on its use during the neonatal period. It can be administered only via the IM route. No serious adverse events have been reported in association with its use^(68)^.

## IRON ISOMALTOSIDE

Iron isomaltoside is not available in Turkey. There are a limited number of studies and inadequate data on its use during pregnancy. Studies revealed adverse event in 1% of patients. The risks associated with its use during lactation are unknown. It can be administered via IV injection and infusion, a test dose is not required^(18,61,69,70,71)^.

## IRON SUCROSE

The use of iron sucrose during pregnancy is approved beginning from the 2^nd^ trimester and is FDA pregnancy category B. Numerous studies on its efficacy and safety, as compared with iron dextran and ferrous gluconate, reported that it was well tolerated; with the exception of urticarial, no hypersensitivity reactions were observed (anaphylaxis, angioedema) and no fatal events were observed. On the other hand, the need for erythropoietin decreases, but Hb, iron, transferrin saturation, serum ferritin, and MCV values increase. Moreover, IV therapy provides a higher serum ferritin value and anemia can be controlled more effectively. The use of iron sucrose during pregnancy, the postpartum period, and the neonatal period has been studied extensively; among iron preparations it has the largest data set^(18,40,72,73,74,75,76)^.

## ADMINISTRATION OF IRON SUCROSE

Iron sucrose usually comes in the form of a 100 mg/5 mL ampoule. It is administered only via the IV route as a slow injection or infusion. During injection, 1 ampoule should be administered over the course of ≥5 min and the dosage per minute should not exceed 20 mg. The maximum dose that can be administered as a single IV injection is 200 mg. In adults a test dose of 20 mg/1 mL should first be administered, if an adverse event is not observed within 15 min the remaining dose should be administered in appropriate time. IV infusion is the preferred method of administration, which reduces the risk of hypotension and paravenous injection. For IV infusion, a 100 mg/5 mL ampoule should be diluted with a maximum of 100 mL of physiologic serum. The administration time should be at least 15 min for 100 mg, 30 min for 200 mg, and should not exceed a total dosage of 200 mg/day. Doses exceeding 200 mg/day are not recommended in pregnancy or the postpartum period because there is not enough safety data^(77)^.

## FERRIC CARBOXYMALTOSE

The use of ferric carboxymaltose in pregnant women has been approved from the 2^nd^ trimester. Studies on IV versus oral iron therapies reported that ferric carboxymaltose was associated with a higher rate of patient tolerability, a higher rate of patient compliance, and target value achievement at lower doses, and that was as safe as but more effective than iron sucrose. With ferric carboxymaltose, a high dose of iron can be administered in a single administration in a short time; hence, problems associated with patient compliance and the additional cost of repeated administrations can be avoided. Ferric carboxymaltose has high molecular stability. Labile iron release or liver necrosis has not been observed in association with its use. Data for and experience with ferric carboxymaltose (pregnancy, postpartum period, neonatal period) are gradually increasing^(18,78,79,80,81,82,83,84,85,86)^.

## ADMINISTRATION OF FERRIC CARBOXYMALTOSE

A 500 mg/10 mL vial of ferric carboxymaltose is administered only via the IV route.

Criteria for determining the total dose of ferric carboxymaltose:


Hb (g/dL)Patient weight 35-70 kgPatients weight >70 kg<101500 mg2000 mg≥101000 mg1500 mgThe total dose should not exceed 500 mg in patients who weigh <35 kg.


The maximum daily dose is 1000 mg/20 mL; because the minimum dose interval is 7 d, this also corresponds to the maximum weekly dose. While administering via injection, the rate of administration should be 100-500 mg/min. Administration time is a minimum of 15 min for doses of 500-1000 mg.

Dilution schedule for administering as infusion:


VolumeIronMaximum volume of physiologic serumMinimum duration of administration2-4 mL100-200 mg50 mL-≥4-10 mL≥200-500 mg100 mL6 min≥10-20 mL≥500-1000 mg250 ml15 min


A placental perfusion study reported that ferric carboxymaltose did not pass to the the fetus via the placenta^(87)^. The FDA approved ferric carboxymaltose in August 2013 and the preparation has since become available as Injectafer; however, to date there has not been a sufficient number of large-scale randomized studies on the use of ferric carboxymaltose during pregnancy and the FDA has specified the pregnancy (2^nd^ and 3^rd^ trimester) category of the drug as C. As such, physicians must carefully consider the risk-benefit ratio when using this drug^(77,88)^.

With regard to international approaches to IV iron therapy, IV iron therapy during pregnancy and the postpartum period in patients with iron deficiency anemia is recommended in the United Kingdom, Germany, Switzerland, Scandinavia, Spain, Eastern Europe, Russia, Pakistan, India, Malaysia, Singapore, Indonesia, China, Thailand, Peru, Argentina, Chile, and Australia. Clear expressions in favor of IV iron are coming from these countries; however, there are many generic drugs on the market, particularly in China, and many studies report that generic iron preparations are less effective and more toxic. Australia strongly recommends IV iron in several indications and is a leading source of publications and guidelines on reducing the need for blood transfusion. Clear expressions about IV iron therapy have not been obtained from the FDA and American College of Obstetricians and Gynecologists (ACOG) from the United States of America^(14,15,16,17,18)^.


Recommendations• IV iron therapy should be included in iron deficiency anemia guidelines published by relevant medical organizations in Turkey, and the principles of iron therapy should be clearly described.


## CONCLUSION

Serious adverse events caused by IV iron preparations that were used in the early years, particularly iron dextran, resulted in concerns about the safety of IV iron therapy. More effective iron preparations have been developed in recent years that are associated with good patient compliance, tolerance, and safety profiles. These preparations are more beneficial than oral iron preparations in some specific indications such as intolerability, low patient compliance, insufficient response to treatment, and prolonged treatment duration. IV iron preparations can be safely used for the treatment of iron deficiency anemia during pregnancy and the postpartum period, particularly for rapid improvement of anemia and rapid replacement of iron storage.

## Figures and Tables

**Table 1:**
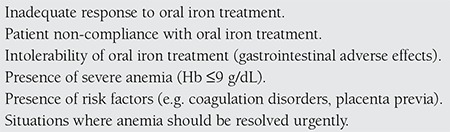
Conditions during pregnancy that require intravenous iron therapy
